# A comparison of intermediate and long-acting insulins in people with type 2 diabetes starting insulin: an observational database study

**DOI:** 10.1111/j.1742-1241.2010.02520.x

**Published:** 2010-11

**Authors:** J Gordon, R D Pockett, A P Tetlow, P McEwan, P D Home

**Affiliations:** 1 School of Population Health and Clinical Practice, University of AdelaideAdelaide, SA, Australia; 2 Centre for Clinical Change and Health Care Research, Flinders UniversityAdelaide, SA, Australia; 3 Cardiff Research Consortium, Eastgate HouseCardiff, UK; 4 Newcastle Diabetes Centre and Newcastle UniversityNewcastle upon Tyne, UK

## Abstract

**Aims:**

Insulin is normally added to oral glucose-lowering drugs in people with type 2 diabetes when glycaemic control becomes suboptimal. We evaluated outcomes in people starting insulin therapy with neutral protamine Hagedorn (NPH), detemir, glargine or premixed insulins.

**Methods:**

Insulin-naïve people with type 2 diabetes (*n* = 8009), ≥ 35 years old, HbA_1c_ ≥ 6.5% and begun on NPH (*n* = 1463), detemir (*n* = 357), glargine (*n* = 2197) or premix (*n* = 3992), were identified from a UK database of primary care records (The Health Improvement Network). Unadjusted and multivariate-adjusted analyses were conducted, with persistence of insulin therapy assessed by survival analysis.

**Results:**

In the study population (*n* = 4337), baseline HbA_1c_ was 9.5 ± 1.6%, falling to 8.4 ± 1.5% over 12 months (change −1.1 ± 1.8%, p < 0.001). Compared with NPH, people taking detemir, glargine and premix had an adjusted reduction in HbA_1c_ from baseline, of 0.00% (p = 0.99), 0.19% (p < 0.001) and 0.03% (p = 0.51). Body weight increased by 2.8 kg overall (p < 0.001), and by 2.3, 1.7, 1.9, and 3.3 kg on NPH, detemir, glargine and premix (p < 0.001 for all groups); insulin dose at 12 months was 0.70 (overall), 0.64, 0.61, 0.56 and 0.76 U/kg/day. After 36 months, 57% of people on NPH, 67% on glargine and 83% on premix remained on their initially prescribed insulin.

**Discussion and Conclusion:**

In routine clinical practice, people with type 2 diabetes commenced on NPH experienced a modest disadvantage in glycaemic control after 12 months compared with other insulins. When comparing the insulins, glargine achieved best HbA_1c_ reduction, while premix showed greatest weight gain and the highest dose requirement, but had the best persistence of therapy.

**What's known**

- Type 2 diabetes is a progressive continuing metabolic disorder in which most people require insulin therapy as endogenous insulin production declines and glycaemic control becomes suboptimal.
- Various insulin preparations are available, each showing differences in molecular structure, pharmacokinetic and pharmacodynamic properties and in clinical outcomes.
- Few studies have made multiple comparisons of different insulin preparations used to begin insulin therapy in people with type 2 diabetes.

**What's new**

- In this observational study of people with type 2 diabetes with suboptimal glycaemic control using oral glucose-lowering drugs and/or lifestyle therapy, significant improvements in glycaemic control were demonstrated after commencing one of four insulin preparations – NPH, detemir, glargine or premix.
- People commenced on NPH insulin had a modest overall disadvantage in outcomes when compared with other insulins.
- Between group comparisons showed that improvements in glycaemic control were greater with insulin glargine, while persistence with therapy was best on premix at a cost of modestly greater weight gain and higher insulin dosage.

## Introduction

Type 2 diabetes is a progressive continuing metabolic disorder characterised by hyperglycaemia caused by insulin deficiency usually on a background of insulin insensitivity (1). As the condition progresses endogenous insulin production declines and most people require insulin therapy (2). Starting insulin results in a clinically relevant improvement in glycated haemoglobin (HbA_1c_) level of around 1.5–3.5% (3,4), and while a number of insulin regimens can be used, clinical decisions about the optimal choice of initial therapy often seem arbitrary (5).

Insulins differ in molecular structure, pharmacokinetic and pharmacodynamic properties, and in clinical outcomes. neutral protamine Hagedorn (NPH) insulin has peak absorption around 4–6 h after injection with a fairly rapid decline thereafter (6), while premixed biphasic insulin (premix) combines basal and short or rapid acting meal-time insulins, the intermediate-acting component being similar to NPH (7). Insulin detemir (detemir) and insulin glargine (glargine) are long-acting human insulin analogues (8,9) providing relatively peak-free insulin levels and longer coverage compared with NPH.

A number of comparative studies have evaluated the various insulin preparations used to begin insulin therapy in people with type 2 diabetes (10–14). The findings in terms of reductions in HbA_1c_, change in body weight and incidence of hypoglycaemia events have varied, while only a few studies have made multiple comparisons of the different treatment options (15,16).

To help resolve some of these issues, and to obtain data from real clinical practice, we have conducted an observational study of changes in glycaemic control, body weight, oral glucose-lowering drug (OGLD) use and insulin dose in people with type 2 diabetes who began one of four insulin types – NPH, detemir, glargine or premix. Our retrospective analysis of a nationwide primary health care database is intended to supplement evidence from prospective non-randomised studies such as PREDICTIVE (17,18). For all insulins except detemir, data were available to 3 years and were used to assess changes over the longer term.

## Database and methods

### Data source

Anonymised data were sourced from a large national (UK) computerised medical record database known as The Health Improvement Network (THIN), which contains longitudinal data collected from UK primary care practices (19). At the time of study, the THIN database included data from 211 practices over a 15-year period, with 2,335,667 people followed prospectively. The THIN database is not supported by any industrial sponsor. Data are collected in routine care during daily record keeping within practices, and are anonymised using encrypted identifiers for the physician and individual.

From data collected between July 2002 and December 2006, as previously described (20,21), 174,094 people were identified with a relevant medical diagnosis (22), or through prescriptions of OGLD therapy. Data were also extracted on diabetic comorbidities. Ethical approval for this study was obtained from the London Multiple Research Ethics Committee (Number 06/MRE02/32) before commencing data extraction.

**Figure 1:**
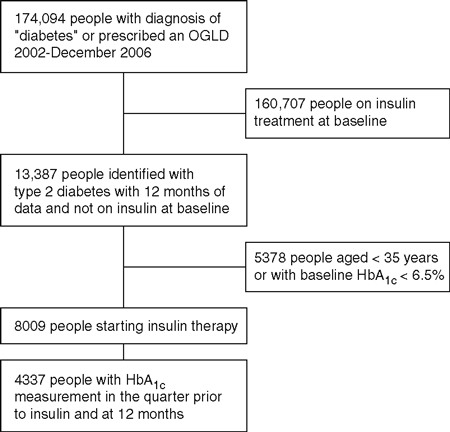
Disposition of participants entering the study from the THIN database. HbA_1c_, glycated haemoglobin; OGLDs, oral glucose-lowering drugs; THIN, The Health Improvement Network

Individuals included in this analysis were required to have not been prescribed insulin within 12 months, been started on NPH, detemir, glargine or premix, with or without meal-time insulin and not have switched to another insulin within 12 months (Figure 1). To reduce the possibility that people with type 1 diabetes would be included in the analysis anyone < 35 years old was excluded, and to reduce insulin starting errors a baseline HbA_1c_ level of ≥ 6.5% was imposed. Use of analogue and human premix insulins could not be distinguished from information collected.

### Design and outcome measures

This was a retrospective, 36-month, non-randomised observational study of prospectively collected data. The principal outcome was pre-defined as change in HbA_1c_ at 12 months. Measurements were performed locally. Although much of the UK is currently HbA_1c_ DCCT-aligned, and primary care practices use National Health Service hospital laboratories which are members of quality assurance schemes, the degree of assay standardisation at the time of data collection (2002–2006) is not known. This study, however, depends on change in HbA_1c_ and will thus be less sensitive to differences in calibration between assays. HbA_1c_ data were analysed for 3-month intervals prior to, and following, beginning insulin therapy using either actual or linearly interpolated values.

Secondary outcomes included change in HbA_1c_ at 36 months, change in body weight, number of prescribed OGLDs over 12 months, daily insulin dose, the use of meal-time insulin and the proportion of people achieving UK specific treatment targets of HbA_1c_≤7.5% and reductions ≥ 1.0% (23). Persistence of use of first prescribed insulin was calculated. Self-reported episodes of hypoglycaemia were recorded by general practitioners (GPs) during each 3 monthly interval.

### Statistical methods

Unadjusted and multivariate-adjusted analyses were conducted. For the unadjusted analyses of change in HbA_1c_, body weight gain and use of OGLDs, patients were required to have an assessment at baseline and data at 12 months or data allowing linear interpolation of such an estimate. Multivariate analysis was used to adjust for baseline characteristics and confounding variables in the analysis of HbA_1c_-related outcomes.

For the unadjusted analyses, linear interpolation of missing data was performed where a patient had at least two data measurements during each 12-month period (prior to and following commencement of insulin) and data were not missing for two consecutive 3-month intervals. For clarity, linearly interpolated data were used to display changes in different outcomes over different time periods.

For change in HbA_1c_ a multivariate analysis, using patient data, was performed using multiple linear mixed regression analyses, adjusted for repeated measures, with change in HbA_1c_ from baseline as the dependent variable and the following pre-defined (fixed-effects) exploratory covariates: baseline HbA_1c_, hypoglycaemia, number of OGLDs, insulin type, mean increase in HbA_1c_ in the 12 months prior to insulinisation, comorbidities prior to commencing the study (pre 2002), concurrent meal-time insulin usage, weight, age, gender, disease duration and associated comorbidities during the study. Differences in the standard of care among general practices were modelled treating practice as a random-effect in the mixed-effects framework; a two-level model was created with patient (level 1) and practice (level 2) as a grouping variable. Treating practice as a random effect explicitly acknowledges that the THIN data are drawn from a larger pool of all practices in the UK. For the results of change from baseline to 12 months overall differences between insulins were analysed, then, where p < 0.05, pairwise comparisons of groups were performed using unpaired *t*-tests.

To adjust for differences in insulin dose between treatments, a standardised measure of glycaemic control was constructed and assessed by multivariate analysis. The ratio of each patient’s mean daily basal insulin dose over 12 months (and 36 months) to the mean basal dose of all people on all treatments was calculated. This ratio was then used to adjust for changes in HbA_1c_, forming the dependent variable in a multivariate model and adjusting for the same set of fixed and random covariates. For the ‘standardised’ basal dose analysis, premix was not included as it contains meal-time insulin.

Sensitivity analyses were performed to investigate the effect of baseline HbA_1c_ levels above 8.0% and 10.0% on the primary outcome. Secondary outcomes (body weight, insulin dose, hypoglycaemia and OGLDs) and the percentage of patients achieving target HbA_1c_ levels were summarised descriptively. Additionally, the proportion of people reaching an HbA_1c_ level of 7.5% and the proportion of people achieving ≥ 1.0% reduction in HbA_1c_ were compared pairwise in logistic mixed-effects models within the generalised linear mixed modelling framework and presented as odds ratios. Persistence on the first prescribed insulin was calculated as a function of duration on that insulin by survival analysis with censoring at the end of the study period.

Multivariate models were developed with SPSS for Windows (version 8; SPSS, Chicago, IL, USA) using a backward stepwise approach. Variables found to be non significant, but that were *a priori* expected to be important in explaining variation in the dependent variable and contributed to a better overall model specification, were kept in the model (24).

## Results

### Study population

A total of 8009 people with type 2 diabetes met the inclusion criteria (Figure 1). Of these, 4337 (54.2%) had an HbA_1c_ measurement at baseline and 12 months. Baseline characteristics for the two populations did not differ materially (Table 1). Premixes were the most commonly prescribed insulins (49.8% of all people), detemir the least (4.5%). The latter insulin was only available for use in the UK since mid 2004. No clinically meaningful differences were found between the therapy groups. Overall glucose control was very poor before commencing insulin (HbA_1c_ 9.5 ± 1.6%). The use of OGLDs was similar across the groups prior to insulin therapy.

**Table 1:** Baseline characteristics of new users of insulin in the quarter prior to commencing insulin therapy for all starters and for those with an HbA_1c_ measurement at baseline and 12 months

Parameter	Detemir	Glargine	NPH	Premix	Total
**All insulin starters**					
People, *n* (%)	357 (4.5)	2197 (27.4)	1463 (18.3)	3992 (49.8)	8009 (100)
Age (years)	58.9 ± 12.1	61.1 ± 12.2	60.7 ± 12.3	61.3 ± 11.6	61.0 ± 11.9
Gender (% male)	47.0	45.0	46.0	43.0	44.0
Body weight (kg)	87.8 ± 22.6	87.4 ± 18.8	88.1 ± 20.1	83.8 ± 18.6	85.4 ± 19.2
Duration of diabetes (years)	5.7 ± 4.9	6.2 ± 5.2	6.1 ± 5.5	6.0 ± 5.6	6.0 ± 5.5
HbA_1c_ (%)§	9.6 ± 1.7	9.5 ± 1.5	9.4 ± 1.6	9.5 ± 1.7	9.5 ± 1.6
OGLDs prescribed, n*	2.02 ± 0.65	2.01 ± 0.62	1.95 ± 0.63	1.92 ± 0.63	1.96 ± 0.63
**With HbA_1c_ result at baseline and 12 months**					
People, *n* (%)†	114 (2.6)	968 (22.3)	727 (16.8)	2528 (58.3)	4337
Age (years)	58.4 ± 11.8	60.1 ± 11.7	59.8 ± 12.4	61.5 ± 11.2	60.8 ± 11.6
Gender (% male)	49.1	45.9	47.3	44.9	45.6
Body weight (kg)	84.9 ± 21.5	87.2 ± 18.9	87.8 ± 21.1	83.7 ± 18.6	85.3 ± 19.3
Duration of diabetes (years)	5.6 ± 4.7	6.4 ± 5.5	5.6 ± 4.7	6.2 ± 5.8	6.2 ± 5.6
HbA_1c_ (%)	9.6 ± 1.7	9.5 ± 1.5	9.4 ± 1.6	9.5 ± 1.7	9.5 ± 1.6
OGLDs prescribed, *n*‡	2.01 ± 0.65	2.03 ± 0.61	2.01 ± 0.65	1.93 ± 0.61	1.96 ± 0.62

Data expressed as mean ± SD unless indicated. Data on ethnic origin were not available. HbA_1c_, glycated haemoglobin; NPH, neutral protamine Hagedorn; OGLDs, oral glucose-lowering drugs.

* Data for OGLDs based on data for 331 people on detemir, 2034 on glargine, 1215 on NPH and 3317 on premix.

† Percentages relate to the proportion of patients in each group of the 4337 patients who had a HbA_1c_ result at baseline and at 12 months.

‡ Data for OGLDs based on data for 109 people starting detemir, 907 on glargine, 556 for NPH and 2528 for premix.

§ Data based on the 4337 patients who had a HbA_1c_ result at baseline and at 12 months.

### Glycated haemoglobin

**Figure 2:**
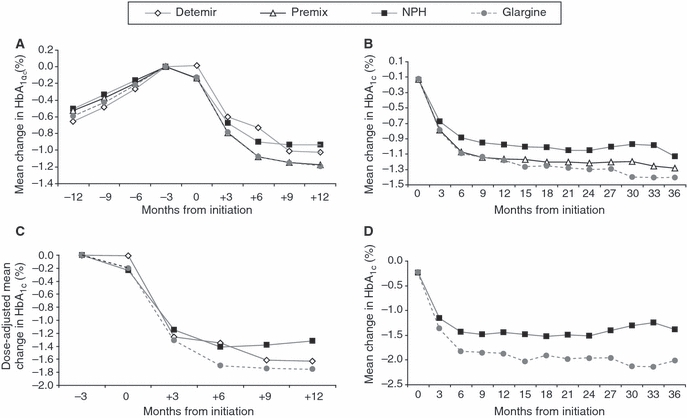
Mean change in HbA_1c_ over 12 and 36 months. The last measurement prior to commencing insulin is at −3 months from initiation. Based on interpolated data. (A) Unadjusted change in HbA_1c_ over 12 months before and after commencing insulin. (B) Unadjusted change in HbA_1c_ over 36 months after commencing insulin based on interpolated data. Data not available for detemir. (C) Dose-adjusted change in HbA_1c_ in the 12 months after commencing insulin. Premix not included as it is a basal–bolus mixture. (D) Dose-adjusted change in HbA_1c_ over 36 months. Premix not included as only basal–bolus mixture. Data not available for detemir. HbA_1c_, glycated haemoglobin; NPH, neutral protamine Hagedorn

Overall, HbA_1c_ increased by 0.56% (from 8.93% to 9.49%) in the 12 months prior to beginning insulin, and fell by 1.1% (from 9.5% to 8.4%, p < 0.001) over the 12 months thereafter (Figure 2A, Table 2). The fall in HbA_1c_ stabilised by 9 months, but with further small reductions over the following 2 years (Figure 2B). By 12 months, unadjusted decrease in HbA_1c_ from baseline was significant in all insulin groups (all p < 0.001). The HbA_1c_ decrease was greatest for glargine and premix (both −1.2%), and least for NPH (−0.9%).

**Table 2:** Unadjusted outcomes for people with type 2 diabetes starting different insulins

Parameter	Detemir	Glargine	NPH	Premix	Total
**Insulin dose at 12 months (U/kg/day)**					
*n* (basal/total)	101/101	896/906	625/673	2317/2380	3939/4060
Basal	0.61 ± 0.48	0.56 ± 0.40	0.64 ± 0.72	0.76 ± 0.54	0.70 ± 0.56
Total*	0.72 ± 0.49	0.66 ± 0.49	0.81 ± 0.88	0.78 ± 0.55	0.76 ± 0.60
**HbA_1c_ (%)**					
*n*	114	968	727	2528	4337
12 months	8.5 ± 1.6	8.3 ± 1.3	8.4 ± 1.5	8.3 ± 1.5	8.4 ± 1.5
Change from baseline	−1.0 ± 2.0†	−1.2 ± 1.7†	−0.9 ± 1.6†	−1.2 ± 1.8†	−1.1 ± 1.8†
**Achieving target (%)**					
≤ 7.5%	28	30	27	33	31
≥ 1.0% reduction	39	57	47	58	55
**Body weight (kg)**					
*n*	101	897	665	2329	3992
12 months	89.5 ± 21.6	89.4 ± 19.1	90.4 ± 21.3	87.1 ± 18.9	88.3 ± 19.5
Change from baseline	+1.7 ± 5.2†	+1.9 ± 6.9†	+2.3 ± 5.2†	+3.3 ± 5.7†	+2.8 ± 6.0†
**OGLDs prescribed, *n***					
*n*	96	792	378	1072	2339
12 months	1.27 ± 0.51	1.31 ± 0.47	1.25 ± 0.45	1.04 ± 0.21	1.17 ± 0.39
Change from baseline	−0.83 ± 0.72†	−0.76 ± 0.66†	−0.82 ± 0.68†	−1.03 ± 0.60†	−0.90 ± 0.65†
**Persistence with therapy, *n* (%)‡**					
Baseline	357	2197	1463	3992	–
12 months	209 (78)	1538 (83)	1042 (75)	3227 (92)	–
24 months	61 (68)	897 (75)	743 (65)	2367 (87)	–
36 months	§	385 (67)	468 (57)	1602 (83)	–
**Use of a meal-time insulin (%)**					
0–12 months	20	14	22	6	12

Data expressed as mean ± standard deviation (SD) or *n* (%) unless indicated. HbA_1c_, glycated haemoglobin; NPH, neutral protamine Hagedorn; OGLDs, oral glucose-lowering drugs.

* Total daily insulin doses include basal plus meal-time insulin

† p < 0.001

‡ Number (%) of people on initial insulin therapy after censoring.

§ Persistence data for detemir were not available at 36 months as the drug was only licensed in the UK from mid 2004.

After adjustment for significant and clinical covariates, there was a significant difference in HbA_1c_ between the groups (p < 0.001) (Table 3). Pairwise comparisons for reductions in HbA_1c_ at 12 months with each insulin vs. NPH were only significant for glargine treatment (−0.19%, p < 0.001). Significantly greater decreases were also observed for the comparisons of glargine vs. detemir and glargine vs. premix; at 36 months, the findings were similar although no data were available for detemir. After dose adjustment (not premix) the findings were similar at both 12 and 36 months with somewhat larger differences (∼0.3%) for the comparisons of glargine vs. NPH or detemir (Figures 2C,D).

**Table 3:** Comparative adjusted reduction in HbA_1c_ in people with type 2 diabetes commencing insulin

	HbA_1c_ reduction (% units)					
	
Parameter	Detemir vs. NPH	Glargine vs. NPH	Premix vs. NPH	Glargine vs. Detemir	Glargine vs. Premix	Premix vs. Detemir
**Change in HbA_1c_ at 12 months**						
Overall	−0.00 ± 0.08	−0.19 ± 0.04†	−0.03 ± 0.04	−0.19 ± 0.07*	−0.16 ± 0.03†	−0.03 ± 0.07
Baseline ≥ 8.0%	−0.02 ± 0.09	−0.25 ± 0.05†	−0.05 ± 0.04	−0.23 ± 0.08*	−0.20 ± 0.04†	−0.03 ± 0.08
Baseline ≥ 10.0%	−0.09 ± 0.15	−0.27 ± 0.09*	−0.01 ± 0.08	−0.36 ± 0.14*	−0.26 ± 0.07†	−0.09 ± 0.14
**Dose-adjusted change in HbA_1c_ at 12 months**						
Overall	−0.01 ± 0.13	−0.30 ± 0.06†	‡	−0.29 ± 0.12*	‡	‡
Baseline ≥ 8.0%	−0.08 ± 0.15	−0.41 ± 0.07†	‡	−0.33 ± 0.14*	‡	‡
Baseline ≥ 10.0%	−0.10 ± 0.25	−0.47 ± 0.14†	‡	−0.37 ± 0.25	‡	‡
Change in HbA_1c_ at 36 months						
Overall	–	−0.12 ± 0.04*	−0.03 ± 0.04	–	−0.09 ± 0.03*	–
Baseline ≥ 8.0%	–	−0.18 ± 0.05†	−0.06 ± 0.05	–	−0.12 ± 0.04*	–
**Dose-adjusted change in HbA_1c_ at 36 months**						
Overall	–	−0.31 ± 0.08†	‡	–	‡	–
Baseline ≥ 8.0%	–	−0.40 ± 0.09†	‡	–	‡	–
Baseline ≥ 10.0%	–	−0.56 ± 0.17†	‡	–	‡	–
**Odds ratio for HbA_1c_reduction [OR (95% CI), p-value]**						
to ≤ 7.5%	1.52 (0.63–2.42), 0.35	1.65 (1.11–2.46), 0.01	1.63 (1.25–2.01), 0.01	1.24 (0.53–2.92), 0.62	0.96 (0.71–1.28), 0.77	1.42 (0.61–3.26), 0.41
≥ 1.0%	0.20 (0.07–0.57), 0.03	1.63 (1.08–2.46), 0.02	1.73 (1.33–2.74), 0.006	3.45 (2.79–4.24), 0.001	0.99 (0.74–1.34), 0.99	4.77 (1.83–12.4), 0.002

Data expressed as mean ± standard deviation (SD) unless indicated. p *<* 0.001 for all between-group differences for all outcomes except unadjusted change at 36 months (p = 0.009). Comparisons with insulin detemir were not possible at 36 months as this insulin was only available in the UK from mid 2004. HbA_1c_, glycated haemoglobin; NPH, neutral protamine Hagedorn.

* p < 0.05.

† p < 0.001.

‡ Analysis not performed as dose adjustment for premix insulin was not possible because of meal-time component.

Sensitivity analyses showed that higher baseline HbA_1c_ levels were correlated with greater reductions over the study periods; for example, the dose adjusted HbA_1c_ change for glargine vs. NPH increased from −0.30 (overall) to −0.47% in people with baseline HbA_1c_ > 10.0% and from −0.29 to −0.37% for the comparison glargine vs. detemir (Table 3).

The proportion of people reaching an HbA_1c_ target of ≤ 7.5% was similar between the insulins (Table 2). Using logistic regression, the odds of reaching that target at 12 months compared with NPH were similar for premix and glargine [odds ratio (OR) 1.63 and 1.65, p = 0.01] (Table 3). Other comparisons were not statistically significant. The odds of achieving reductions in HbA_1c_≥ 1.0% were similar with glargine and premix, both of which were significantly greater compared with NPH and detemir.

### OGLD usage and insulin dose

**Figure 3:**
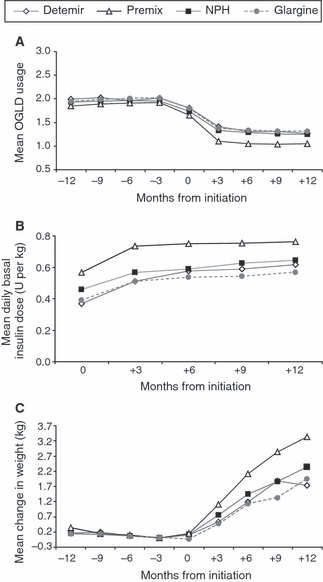
(A) Mean OGLDs prescribed. (B) Mean change in daily basal insulin dose (U/kg) over 12 months. (C) Mean weight change in 12 months prior to and after commencing insulin (unadjusted data). HbA_1c_, glycated haemoglobin; NPH, neutral protamine Hagedorn; OGLD, oral glucose-lowering drug. The last measurement prior to commencing insulin is at −3 months

The number of concomitant OGLDs used fell (p < 0.001) with insulin treatment, from overall mean of 1.96 in all people (individual insulin range 1.92–2.02) per day at baseline (Table 1) to mean of 1.17 (1.04–1.31) at 12 months; a change of between −0.76 types/day with glargine and −1.03 types/day with premix (Table 2, Figure 3A).

Following initiation, basal insulin doses increased gradually over the year, but mostly in the first 3 months (Figure 3B). Total daily insulin doses, including meal-time insulin, were lower with glargine (0.66 ± 0.49 U/kg) and highest with NPH insulin (0.81 ± 0.88 U/kg) (Table 2). The proportion of people receiving meal-time insulin over the 12 months NPH, detemir or glargine was 22% for NPH, 20% for detemir and 14% for glargine.

### Body weight and hypoglycaemia

People generally gained weight on all regimens (p < 0.001 overall), with a weight (kg) increase at 12 months; ranging from 1.7 ± 5.2 (SD) kg on detemir, 1.9 ± 6.9 kg on glargine, 2.3 ± 5.2 kg on NPH and 3.3 ± 5.7 kg on premix (Table 2, Figure 3C).

People who were on oral agents during the 12 months prior to commencing insulin reported 645 hypoglycaemic episodes (0.11 events per patient-year); 0.08 episodes per patient/year for NPH, 0.10 for premix, 0.13 for glargine, and 0.14 for detemir. Reported events per patient-year remained low during the 12 months after starting insulin: 0.12 for detemir, 0.14 for NPH, 0.18 for glargine and 0.25 events per patient-year for premix.

### Persistence

**Figure 4:**
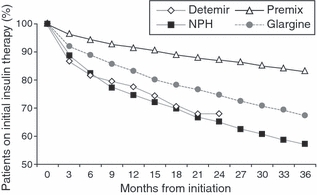
Percentage of patients remaining on initial type of insulin therapy. From initiation, the number of quarters, patients remain on their initially prescribed insulin, was calculated (this varied by patient, some remained on their initially prescribed insulin for 6 months, others 12 months etc.). Those patients remaining on their initially prescribed insulin at the end of their recorded history were censored (removed from the calculations) at the end of the recorded history period. The changes in HbA_1c_ at 36 months were then calculated against these quarters. NPH, neutral protamine Hagedorn

After 12 months, 75% of people on NPH remained on the insulin, compared with 78% for detemir, 83% for glargine and 92% for premix (Table 2, Figure 4). At 36 months, this order was unchanged (no data for detemir), with significant between group differences (p < 0.001). Pairwise comparison at 36 months showed premix persistence was better than for other insulins (all p < 0.001). At 24 months, glargine persistence was better than detemir (p = 0.014) and at 36 months it was better than NPH (p < 0.001).

## Discussion

This non-interventional study assessed clinical outcomes in people with type 2 diabetes starting insulin therapy using each of the four usual insulin preparations. Baseline HbA_1c_ was well above recommended thresholds for starting insulin (4,5), as has been well recognised in other studies (15,17). Part of the reason for this was a rapid deterioration in blood glucose control in the year before starting therapy. At 12 months, insulin therapy was an effective strategy in reducing HbA_1c_ in a population with a very diverse duration of diabetes (SD 5–6 years), but the reduction of a little over 1.0% is somewhat disappointing compared with treat-to-target studies (14,25), despite similar insulin doses at 1 year. However, it is reassuring that the improvement in glycaemic control was maintained over the 3 years of study. The reduction in use of concomitant OGLDs indicates that additional use of these treatments may have been associated with further improvement in glycaemic control.

Those begun on NPH insulin had rather lesser improvements in HbA_1c_ than for the other insulins. However, after adjustment for other variables, NPH and premix did not differ at 12 or 36 months, but the overall change with insulin glargine was about 0.2% units better at 12 months compared with NPH, premix and detemir. This effect was larger in people with higher baseline HbA_1c_ levels. Comparisons with detemir are less certain because of small number of patients on this treatment, but the analysis suggested that this too did not differ from NPH insulin. Incorporating dose adjustment in the analysis showed improved glycaemic control with glargine compared with both NPH and detemir.

In general, improvements in blood glucose control can only be judged in the context of hypoglycaemia incidence as well as insulin dose. Here our data are unhelpful. The hypoglycaemia data in the THIN database rely on people self-reporting events to their GPs, and it is clear that even allowing for the relative hyperglycaemia, the event rates reported here are likely to be severe underestimates of what is normally observed. Another plausible explanation for the low rate of hypoglycaemia is that some patients may not have received adequate titration of their insulin dose by their primary care physicians in accordance with recommended levels, as indicated by the modest reduction in HbA_1c_ levels observed in the study.

Body weight gain on starting insulin therapy is usually resulting from amelioration of urinary glycosuria and of glucose concentration driven glucose metabolism (26). That the smallest weight gain was with detemir, and the greatest with premix insulin, is consistent with previous reports from randomised studies (14,25,27). However, the wide variation (SD ± 5–7 kg) indicates a very diverse experience among people on any one insulin, considerably dwarfing the 1.5 kg mean difference between the lowest and the highest insulin groups.

The pattern of persistence with the different treatments did not accord with any of the above variables associated with diabetes control. Persistence at 1, 2 and 3 years was greatest with premix, intermediate with glargine and least with NPH. Data on detemir were limited, but appeared to be similar to that of NPH in years 1 and 2.

Nevertheless, the detemir data allow some check on the validity of the results when randomised controlled trials and observational studies are compared. In the treating to target in type 2 diabetes (4T) trial (15) detemir was also less effective than premix at controlling HbA_1c_ after 1 year of treatment, but with lower insulin doses and with less weight gain. After 3 years of treatment, glycaemic control was similar between the different insulin groups, although detemir required higher doses than premix but had a lower rate of hypo-glycaemia (16). Compared with patients initiating detemir in the PREDICTIVE observational study (17,18), improvements in glycaemic control in our study were more modest (1.0% vs. 1.3%) with an increase in body weight of 1.7 kg compared with a decrease of 0.5–0.9 kg observed in the PREDICTIVE cohort. These differences are likely to be attributed to various factors, including the manner in which subjects are recruited into each study cohort (e.g. enrolment of patients from specialised vs. primary practice centres) and the degree of insulin titration employed. However, as a result of the low number of subjects in our study who were started on detemir, the results should be interpreted with caution. Interestingly, in forced titration studies where people are treated to a glucose target, glucose control tends to be similar between NPH insulin and insulin glargine, but hypoglycaemia rates are very different (by ∼40%) (28). In our study, in which hypoglycaemia cannot be reliably assessed, glucose control was better with insulin glargine compared with NPH as might be expected if experience of hypoglycaemia limits insulin dose titration in regular clinical practice.

This observational study has a number of strengths. It is a population-based assessment of a large number of people with documented baseline characteristics over 12 months prior to starting of insulin. Available data over 12 months and up to 3 years allowed assessment of outcomes over the medium term and for a period when changes are likely to be maximal. Importantly, the 3-year data provides assurance that changes at 12 months were valid indicators of effects in the longer term, of importance in a life-long chronic condition. This is enhanced by the completeness of the follow-up assessments over that time. Furthermore, the dataset is reasonably comprehensive for all of the important variables, including use of oral glucose-lowering agents as well as insulin dose.

An important aspect here is that the insulin doses here are derived from real clinical practice, and not influenced by any algorithm imposed on the study, nor even the knowledge of being part of a study (study effect). Accordingly, the results should be a true reflection of the differences between the different insulins in clinical practice, and able to be generalised for the purposes of making policy decisions on provision of diabetes care, feeding economic models and reviewing the overall performance of UK care in this area.

The study does have limitations. Perhaps the most important is that, like all observational studies, unknown biases may determine which people get which type of insulin. Both insulin glargine and particularly insulin detemir were relatively new to the market at the time of study, and it might be imagined on the one hand that early adopters among physicians would be keener on better blood glucose control, but on the other that lesser experience of the new insulins would limit their optimal use. Since the data were collected from a large number of primary practice units with resulting differences in glycaemic targets, level of care and physician experience, this is likely to introduce a level of heterogeneity to the main findings. We attempted to account for this by treating the practice as a random-effect in the mixed-effects framework.

Another issue is that only 54% of people in the main study population had measurements at baseline and at 12 months, despite the use of linearly interpolated data. However, as the baseline characteristics for this population did not differ compared with the overall study population, it is likely that the results are applicable to the main study population.

Some data are not reliably collected in databases like THIN. Symptomatic hypoglycaemia relies on patient recall, may not be enquired after, and may not be recorded unless a self-monitored result is available or medical-paramedical support is required. The event rates in this study are below background rates recorded in randomised controlled trials, and although the higher rate on premix is consistent with the 4T study the absolute rates are probably best ignored (15,16). They are reported only for completeness.

Within the THIN database, we are unable to evaluate outcomes in people receiving human vs. analogue premix insulins. At the time of study the likely mix of human and analogue insulins represented by our category ‘premix’ would be similar to the UK market at the time, namely 44/56% respectively (29).

Finally, our study cannot determine the reasons for not achieving better control than mean HbA_1c_ levels still > 8.0% after 12 months. It is noteworthy that some sectors of UK primary care remained unconvinced of the benefits of blood glucose control despite evidence-based reviews and meta-analyses (30). A problem here is that it cannot be assumed that the differences found between the insulins in attaining the levels of control found in the current study would also be found were real clinical practice to attain better results. Nevertheless, as the results are consistent with the 1-year findings of the 4T study (15), and as the differences in hypoglycaemia rates tend to be greater at lower HbA_1c_ levels in the randomised studies (31), it is a reasonable assumption that the differences between the insulins described here would be no smaller with tighter control.

Despite these shortcomings, observational studies such as the current report are generally regarded as an ideal approach to assess the actual health outcomes of patients in routine care. This is because the level of care patients receive in clinical trials is often of a higher standard and not representative of that provided in daily clinical practice. There are recognised trade-offs between prospective studies designed to be internally valid (i.e. randomised controlled trials) and studies aimed at characterising outcomes observed in actually treated patients (i.e. observational studies) – each study type adds something to the evidence base and we have followed accepted procedures (i.e. inclusion/exclusion criteria, statistical methodology) in minimising the well-known biases/deficiencies of database studies.

In summary, in real clinical practice in the UK, in people with suboptimal glycaemic control with OGLDs and lifestyle therapy, insulin is an effective strategy in reducing HbA_1c_ levels. People commenced on NPH have a modest overall disadvantage in outcomes when compared with other insulins. Between group comparisons showed that HbA_1c_ reductions were greater with insulin glargine, while persistence with therapy was best on premix at a cost of modestly greater weight gain and higher insulin dosage.
